# Supplementary material for: Roles of sonic hedgehog signaling in retinal patterning and neurogenesis during mammalian eye development
Source: Development. 2026 May 12;153(9):dev205143. doi: 10.1242/dev.205143 (PMC13245920; doi:10.1242/dev.205143)
Supplement: Supplementary information [file develop-153-205143-s1.pdf]

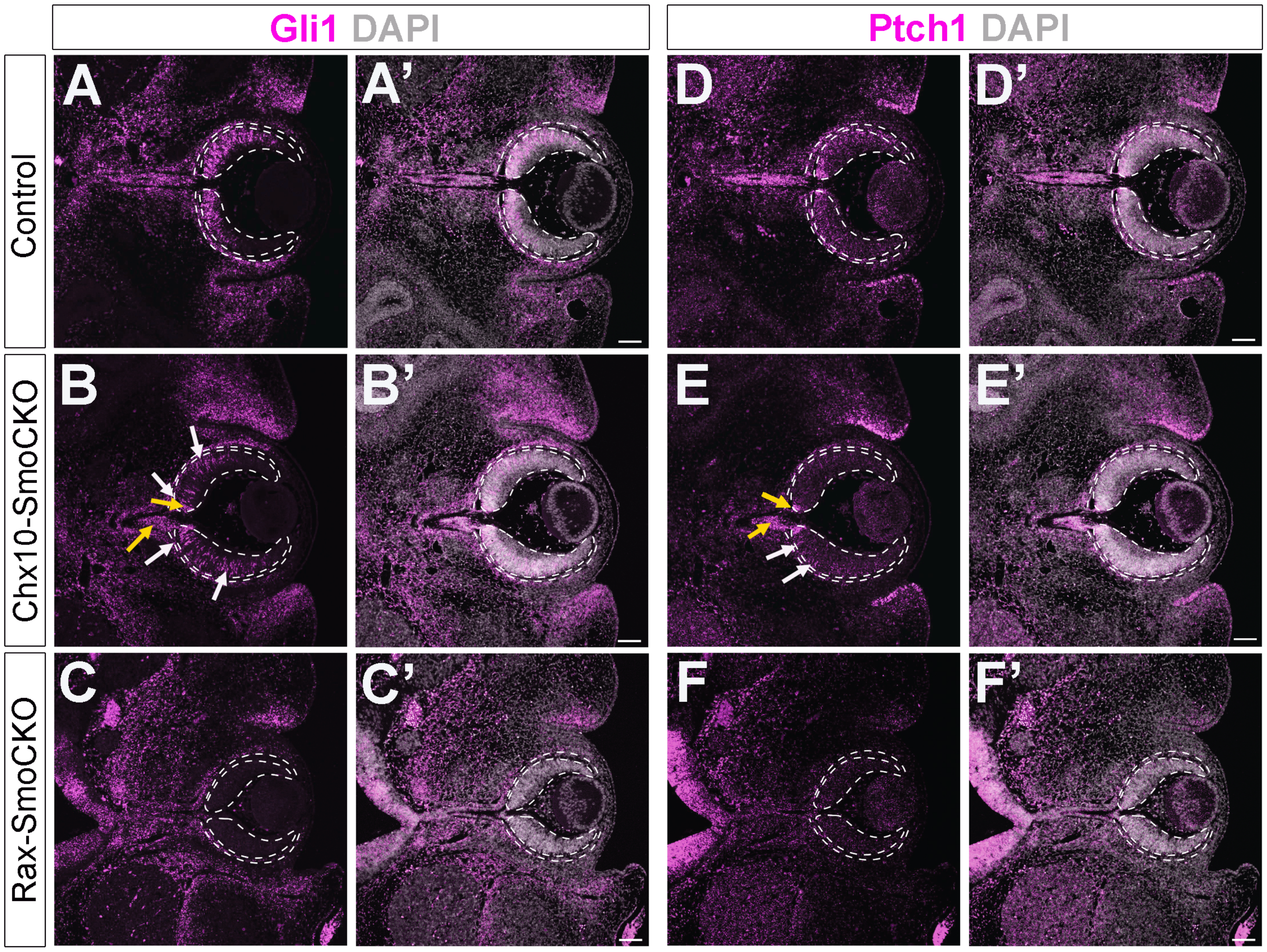

Fig. S1. Downregulation of *Gli1* and *Ptch1* upon *Smo* ablation.

A-C') RNAscope in situ hybridization of *Gli1* (magenta). **D-F')** RNAscope in situ hybridization of *Ptch1* (magenta). In all cases, the tissue was counterstained with DAPI (gray, A'. B'. C', E', E', F'). Note the mosaicism of the Chx10-SmoCKO model reflected in patches of *Gli1*+ and *Ptch1*+ in RPCs (white arrows) and high levels of both *Gli1* and *Ptch1* in the ONH and optic stalk (yellow arrows). Scale bar: 100 microns

Development • Supplementary information

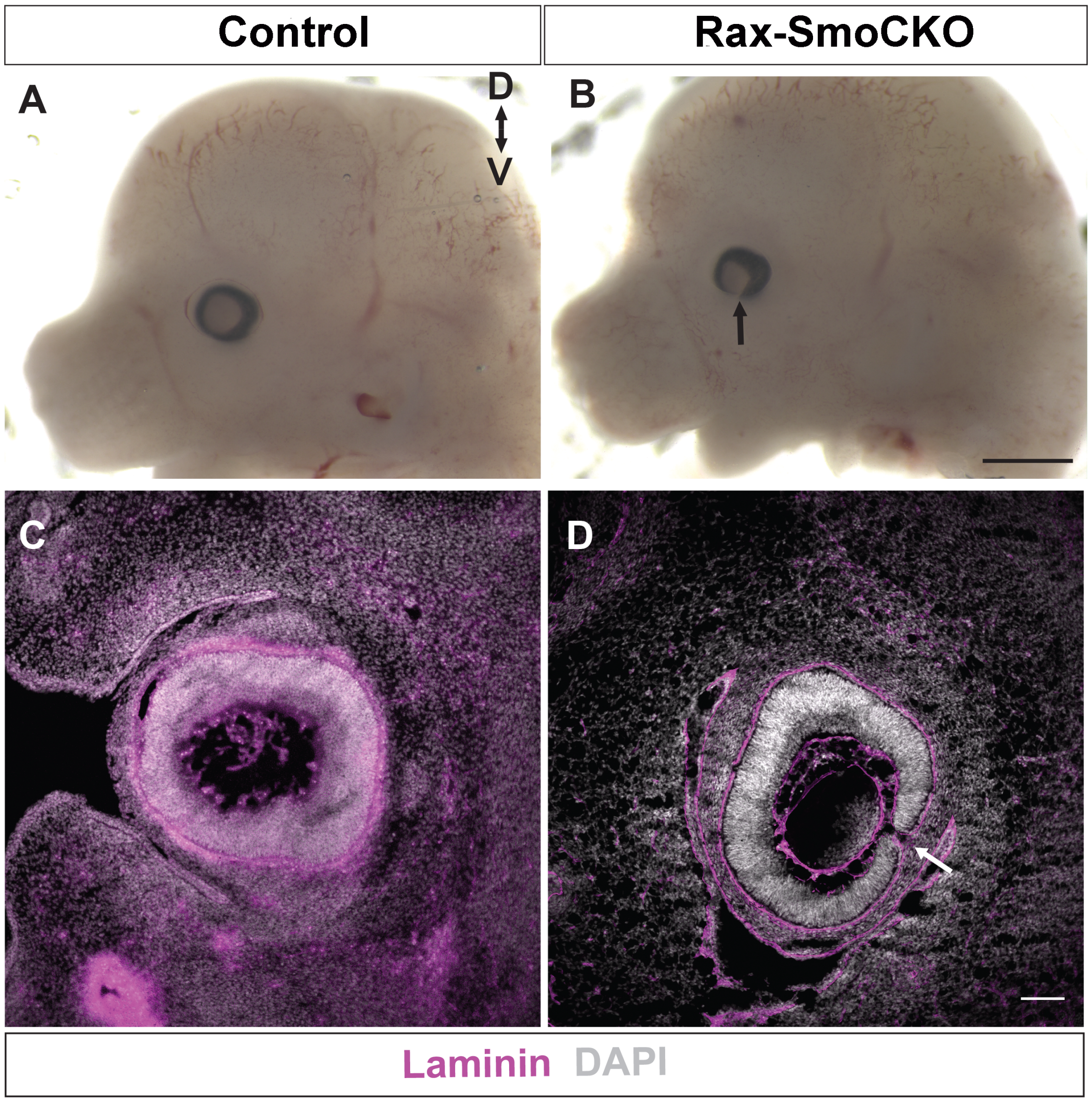

Fig. S2. Only Rax-SmoCKO mice exhibit coloboma

A-B) Photomicrographs of control and Rax-SmoCKO mice at E13.5. Note the coloboma in B (arrow). **C-D)** Sagital sections of E13.5 samples stained with Laminin (magenta) and DAPI (gray). Note the gap in the ventral part of the optic cup in in D (arrow). Scale bars: 1.5mm in A-B, 100 microns in C-D. For these experiments, we examined n = 12 Rax-SmoCKO mutants, n =7 Chx-SmoCKO mutants and n=17 WT eyes.

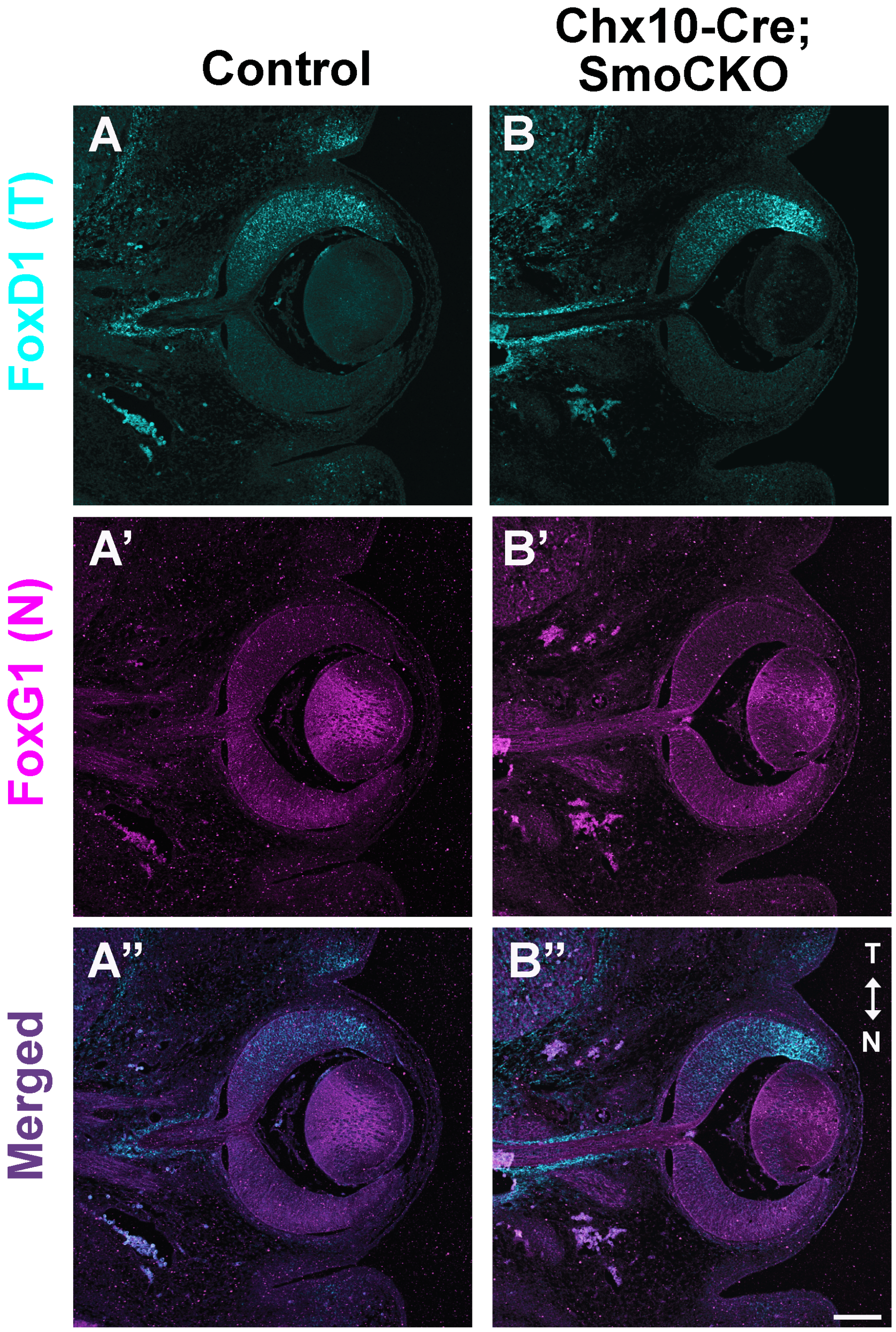

Fig. S3. Nasal-temporal patterning in Chx10-SmoCKO mice.

Horizonal sections with *FoxG1* (teal, **A-A'**) and *FoxD1* RNA ISH (magenta, **B-B'**) at E13. Scale bars: 100 microns.

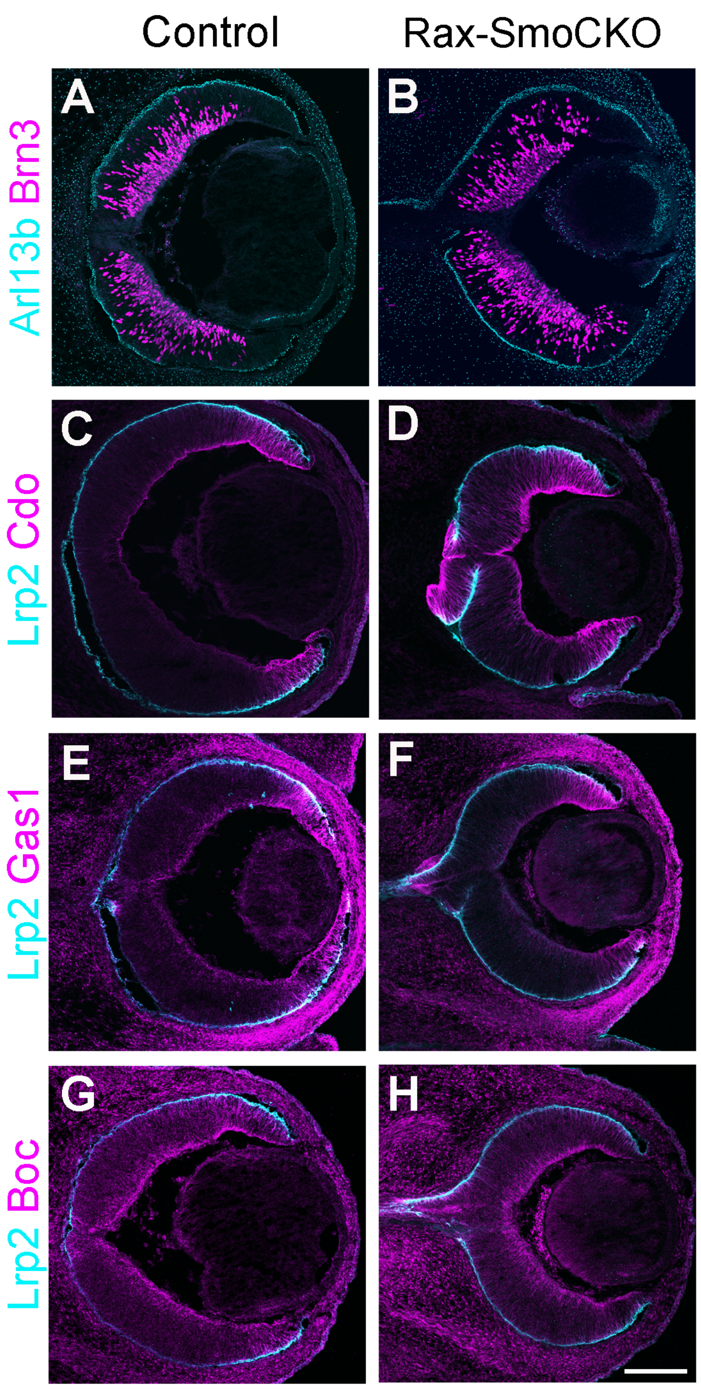

Fig. S4. *Smo* ablation did not affect primary cilia position, nor Shh co-receptor expression.

Immunostaining for Arl13b (teal), a protein enriched in primary cilia, and the RGC marker Brn3 (magenta, **A-B**). The expression of Shh co-receptors were directly compared by labeling with Lrp2 (teal) and Cdo (magenta, **C-D**), Lrp2 (teal) and Gas1 (magenta, **E-F**), and Lrp2 (teal) and Boc (magenta, **G-H**) in E13.5 eyes. Scale bar: 200 microns.

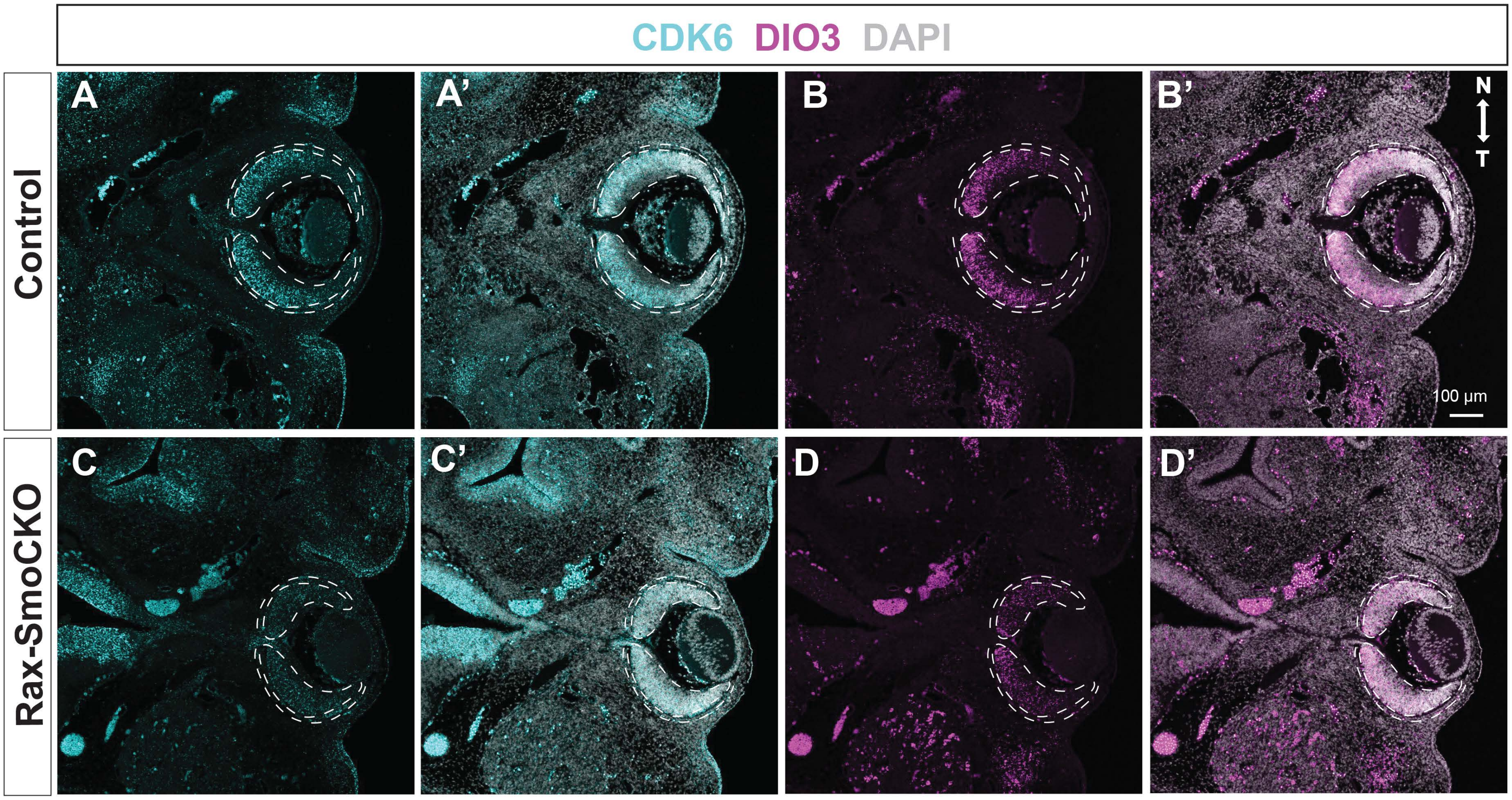

Fig. S5. *Smo* ablation reduced Dio3 and Cdk6 mRNA expression in E13.5 retinas.

In situ hybridization (RNAscope) for *Cdk6* (teal, **A-A' and C-C'**) and *Dio3* (magenta, **B-B' and D-D'**) are shown. All the samples were counterstained with DAPI (gray). Scale bar: 100 microns.

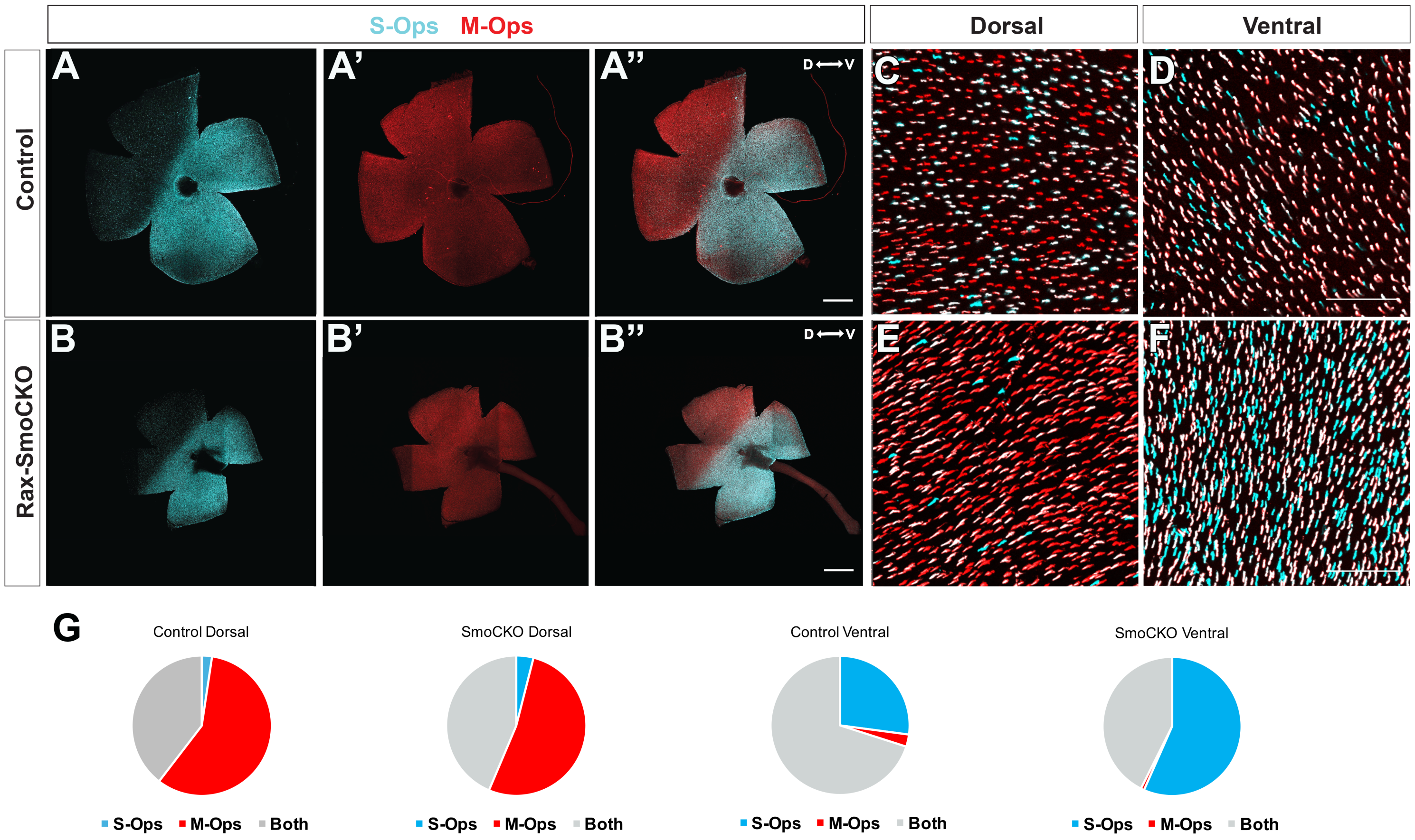

Fig. S6. At P21, Rax-SmoCKO retinas exhibited higher ratios of cone photoreceptor cells and changes in the proportions of cone subtypes in the ventral retina.

A-B'') Flat-mounted retinas stained with Blue-Opsin (S-Ops, teal) and Red/green-Opsin (M-Ops, red). **C-F)** Close-up images of the outer segments in the different regions. **G)** Quantifications of the ratios of cone subtypes, classified as shown in (Nadal-Nicolas et al., 2020). Note the increase in true S-cones in the ventral region of the Rax-SmoCKO retina. Scale bars: 100 microns for A-B'', 50 microns for C-F

Development • Supplementary information

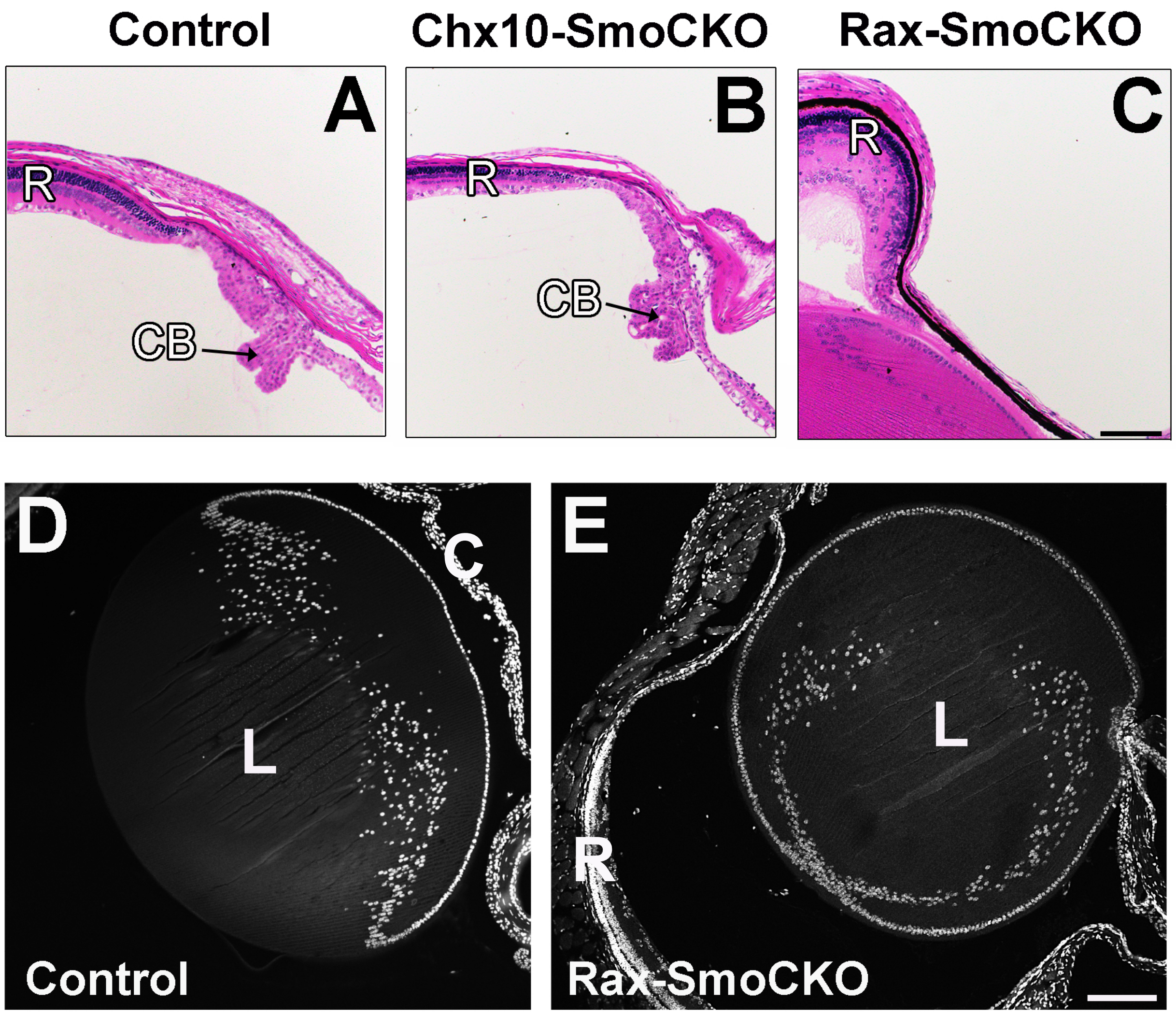

Fig. S7. Rax-SmoCKO mice have defective lenses and lack ciliary bodies. A-C) H&E staining of the different mouse lines showing the presumptive absence of ciliary body in Rax-SmoCKO eyes but not in Chx10-SmoCKO. **D-E)** DAPI staining of control and Rax-SmoCKO lens. R: retina, CB; ciliary body, L: lens, C: cornea. Scale bars: 100 microns in A-C, 250 microns in D-E.

Development • Supplementary information

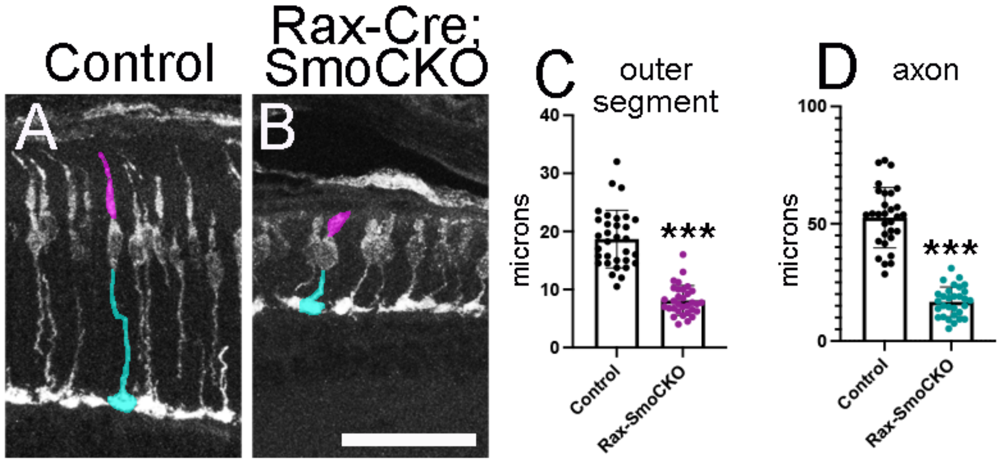

Fig. S8. Cone photoreceptors show altered morphologies at P21 in Rax-SmoCKO mice.

A-B'') P21 sections stained with Cone Arrestin (ConeArr, white) show shortened axon length in Rax-SmoCKO mice **C-D)** Quantification of average outer segment and axon lengths in microns. ***p<0.005. Scale bar: 50 microns.

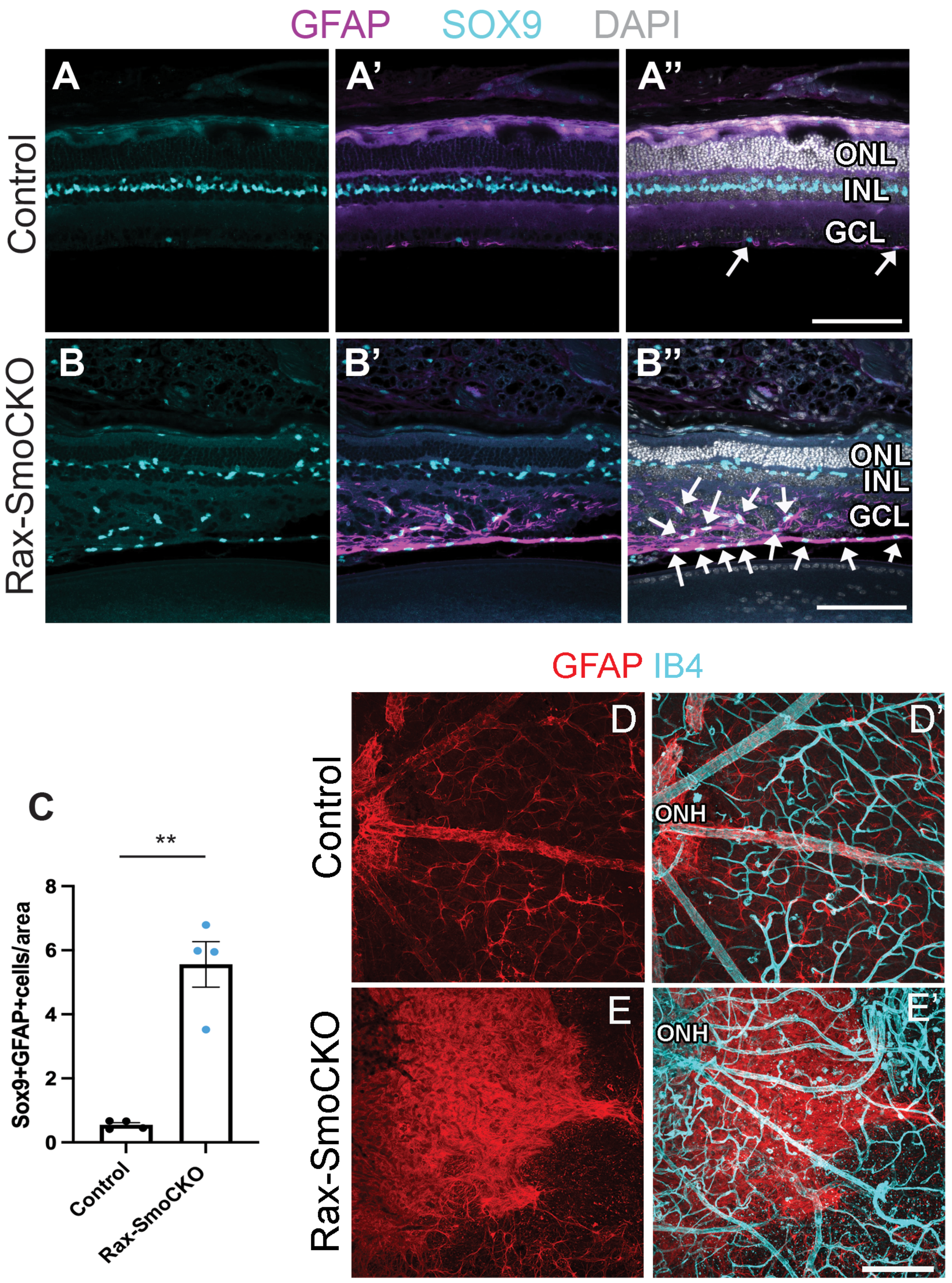

Fig. S9. Rax-SmoCKO mice yield increased number of retinal astrocytes and altered vasculature.

A-B’’) P21 retinas stained with Sox9 (teal) and GFAP (magenta), and counterstained with DAPI (gray) show increased number of Sox9+ GFAP+ retinal astrocyte (arrows). **(C)** Quantification of Sox9+GFAP+ astrocytic cells, **p=0.0019. **(D-E)** P21 flat-mounted retinas were stained with GFAP (red) and Isolectin-IB4 (teal). Note the aberrant morphologies in E’. Scale bars: 100 microns.

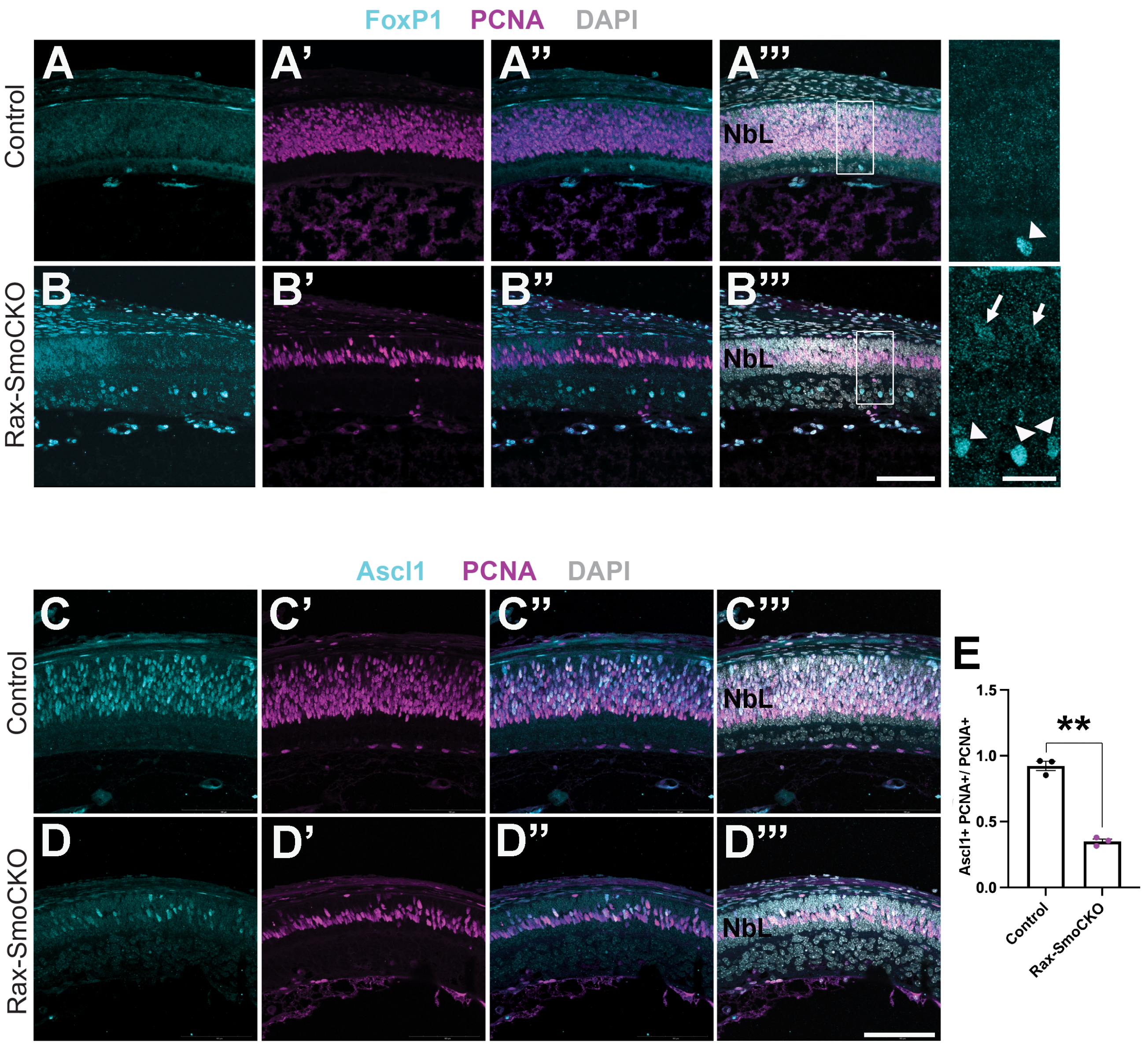

Fig. S10. Rax-SmoCKO mice exhibit altered expression of early and late transcription factors at P1. (A-B’’) Staining with FoxP1 (teal) and PCNA (magenta) show increased levels of FoxP1 in Rax-SmoCKO RPCs (arrows). Please note that FoxP1 is also expressed in a subpopulation of RGCs (FoxP1+ cells located in the GCL, arrowheads). **(C-D’’)** Staining with Ascl1 (teal) and PCNA (magenta) show less Ascl1+ PCNA+ cells in Rax-SmoCKO mice as quantified in **(E)** ***p=0.0007**.** Scale bars: 100 microns.

Development • Supplementary information

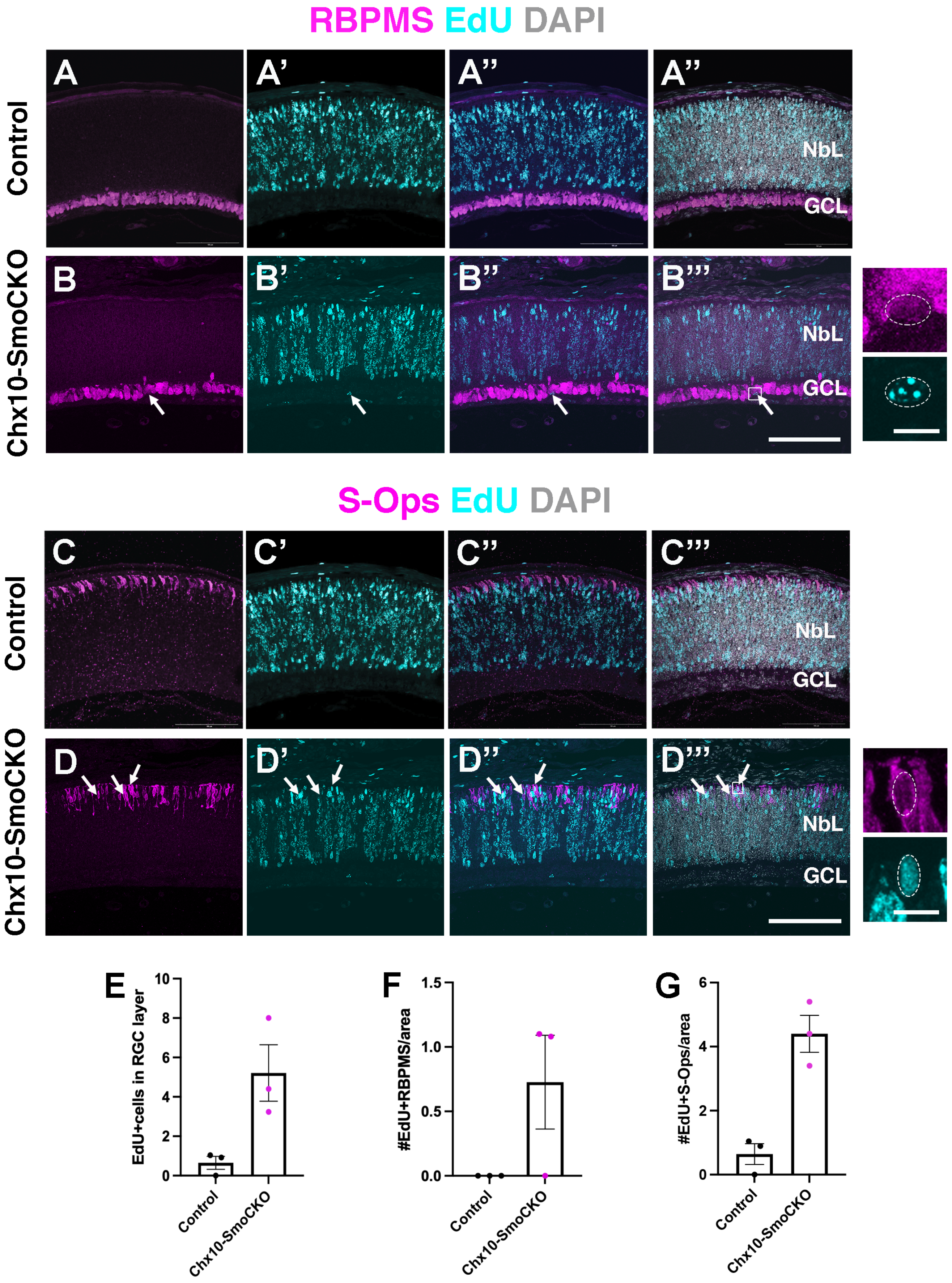

Fig. S11. Neurogenesis timing is altered in Chx10-Cre; SmoCKO retinas. Control and Chx10-SmoCKO mice were injected with EdU at E17 to label RPCs and samples were collected at P2. **(A-B''')** Co-staining with RBPMS (magenta), EdU (teal), and DAPI (gray) revealed EdU+ RBPMS+ cells in the central retinas of Rax-SmoCKO mice (arrows) but not in controls. A magnification of the area indicated in B''' is shown in the right-most panels. **(C-D''')** Co-staining with S-Opsin (magenta), EdU (teal), and DAPI (gray). Arrows show EdU+ S-Opsin+ cells. **E-G)** Quantification of number of EdU+ cells in GCL, EdU+ RPMBS+ cells, and EdU+ S-Opsin+ cells, respectively. All datapoints are normalized by area. NbL: Neuroblastic layer, GCL: Ganglion Cell layer. Scale bar: 100 microns and 10 microns in the insets.

Development • Supplementary information

Table S1. RNA-Sequencing expression data from control and Rax-SmoCKO E13.5 eyes. Normalized RNA-seq counts for genes detected in four control (CONTROL1-4) and four Rax-SmoCKO (SMO1-4) samples.

Available for download at
https://journals.biologists.com/dev/article-lookup/doi/10.1242/dev.205143#supplementary-data

Table S2. Primary Antibodies

Antibody	Source	Catalog	Concentration	RRID
Anti-Arl13b (Rabbit)	Proteintech	17711-1AP	1:1000	AB_2060867
Anti-Atoh7 (Rabbit)	Novus	NBP1-88639	1:500	AB_11034390
Anti-AP2-alpha (Mouse)	Thermofisher	3B5	1:200	AB_2199412
Anti-Ascl1	Abcam	AB211327	1:200	AB_2924270
Anti-Boc (Goat)	R&D Systems	AF2385	1:500	AB_2066909
Anti-BRN3 (Goat)	Santa Cruz	sc-6026	1:100	AB_673441
Anti-Calbindin (Mouse)	Neuromab	73-452	1:250	AB_2629417
Anti-Calretinin (Goat)	Swant	CG1	1:200	AB_10000342
Anti-Cdo (Goat)	R&D Systems	AF2429	1:500	AB_2078891
Anti-Cone Arrestin (Rabbit)	Millipore	Ab15282	1:400	AB_1163387
Anti-CRX (Mouse)	Abnova	H00001406-M02	1:200	AB_538009
Anti-FoxG1 (Rabbit)	Abcam	Ab196868	1:200	AB_2892604
Anti-FoxP1	Cell Signaling	2005	1:200	AB_2106979
Anti-Gas1 (Goat)	R&D Systems	AF2644	1:500	AB_2107951

Development • Supplementary information

Anti-GFAP (Mouse)	Abcam	Ab7260	1:100	AB_305808
Anti-Glutamine Synthetase (Mouse)	Millipore	MAB302	1:100	AB_2110656
Ib4 lectin	Life Technologies	I21411	1:100	AB_2314662
Anti-Laminin (Rabbit)	Abcam	AB11575	1:500	AB_298179
Anti-Lrp2 (Rabbit)	Abcam	AB76969	1:1000	AB_10673466
Anti-Opsin Red/Green (Rabbit)	Millipore	AB5405	1:200	AB_177456
Anti-Opsin Blue (Goat)	Santa Cruz	sc-14363	1:200	AB_2158332
Anti-OTX2 (Goat)	R&D Systems	AF1979	1:200	AB_2157172
Anti-PAX2 (Rabbit)	Biolegend	901001	1:1000	AB_2565001
Anti-PAX6 (Mouse)	Santa Cruz	sc-32766	1:50	AB_628107
Anti-PCNA (Mouse)	Invitrogen	13-3900	1:200	AB_86593
Anti-PH3 (Rabbit)	Thermo Fisher	PA5-17869	1:100	AB_10984484
Anti-RBPMS (Rabbit)	Proteintech	15187-1-AP	1:400	AB_2238431
Anti-SOX2 (Goat)	R&D Systems	AF2018	1:100	AB_355110
Anti-SOX9 (Rabbit)	Millipore	AB5535	1:200	AB_2239761
Anti-TUJ1 (Mouse)	BioLegend	801201	1:500	AB_2313773

CONFIDENTIAL

Table S3. Secondary antibodies

Antibody	Source	Catalog	Concentration
Alexa fluor 488 donkey anti-mouse	Invitrogen	A21202	1:200
Alexa fluor 568 donkey anti-mouse	Invitrogen	A10037	1:200
Alexa fluor 647 donkey anti-mouse	Invitrogen	A31571	1:200
Alexa fluor 488 donkey anti-rabbit	Invitrogen	A21206	1:200
Alexa fluor 568 donkey anti-rabbit	Invitrogen	A10042	1:200
Alexa fluor 647 donkey anti-rabbit	Invitrogen	A31573	1:200
Alexa fluor 488 donkey anti-goat	Invitrogen	A11055	1:200
Alexa fluor 568 donkey anti-goat	Invitrogen	A11057	1:200
Alexa fluor 647 donkey anti-goat	Invitrogen	A21447	1:200
