# Supplementary material for: Roles of sonic hedgehog signaling in retinal patterning and neurogenesis during mammalian eye development
Source: Development. 2026 May 12;153(9):dev205143. doi: 10.1242/dev.205143 (PMC13245920; doi:10.1242/dev.205143)
Supplement: Supplementary file 1 [file develop-153-205143_review_history.pdf]

Development

Roles of sonic hedgehog signaling in retinal patterning and neurogenesis during mammalian eye development

Miranda R. Krueger, Simranjeet K. Cheema, Sergi Simo, Edward M. Levine, Nadean L. Brown and Anna La Torre
DOI: 10.1242/dev.205143

Editor: James Briscoe

Review timeline
Original submission: 31 July 2025
Editorial decision: 2 September 2025
First revision received: 10 March 2026
Accepted: 8 April 2026

Original submission

First decision letter

MS ID#: dev.205143

MS TITLE: Novel Roles of Sonic Hedgehog Signaling in Retinal Patterning and Neurogenesis During Mammalian Eye Development

AUTHORS: Miranda R. Krueger, Simranjeet K. Cheema, Sergi Simo, Edward M. Levine, Nadean L. Brown and Anna La Torre

Dear Dr La Torre,

I have now received all the referees' reports on the above manuscript, and have reached a decision. The referees' comments are appended below, or you can access them online: please go to: View Reviewer Comments

As you will see, the referees express considerable interest in your work, but have some significant criticisms and recommend a substantial revision of your manuscript before we can consider publication. The referees would like additional mechanistic data on temporal competence regulation. This could include analysis of competence factors such as Ikaros and examination of early/late temporal identity markers beyond Atoh7. The temporal competence phenotype should be validated using the Chx10-Cre driver to distinguish direct versus secondary effects of Hedgehog signaling. Please also moderate claims of novelty, particularly regarding optic nerve head specification, and provide appropriate citations to prior work. Additional quantitative analysis is needed for astrocyte numbers and morphological claims about cone photoreceptors require higher magnification images and polarity marker staining for support.

If you are able to revise the manuscript along the lines suggested, which may involve further experiments, I will be happy to receive a revised version of the manuscript. Your revised paper will be re-reviewed by one or more of the original referees, and acceptance of your manuscript will depend on your addressing satisfactorily the reviewers' major concerns. Please also note that Development will normally permit only one round of major revision. If it would be helpful, you are welcome to contact us to discuss your revision in greater detail. Please send us a point-by-point response indicating your plans for addressing the referees' comments, and we will look over this and provide further guidance.

© 2026. Published by The Company of Biologists under the terms of the Creative Commons Attribution License (https://creativecommons.org/licenses/by/4.0/).

Please attend to all of the reviewers' comments and ensure that you clearly highlight all changes made in the revised manuscript. Please avoid using 'Tracked changes' in Word files as these are lost in PDF conversion. I should be grateful if you would also provide a point-by-point response detailing how you have dealt with the points raised by the reviewers in the 'Response to Reviewers' box. If you do not agree with any of their criticisms or suggestions please explain clearly why this is so.

Reviewer 1

Advance summary and potential significance to field

This manuscript describes the effects of conditional loss of function of Smoothened (Smo), and by consequence Shh/Ihh signaling, in Rax-positive ocular neuroepithelial cells. While previous studies had characterized the effects of disrupting Smo in Vsx2-positive retinal progenitor cells, the current study describes the phenotype of a substantially earlier loss of function. This leads to dramatic changes in retinal patterning, including conversion of optic nerve into neuroretina, breakdown of anterior-posterior patterning, and loss of ciliary margin. Precocious neurogenesis and changes in temporal patterning are also observed, which are substantially stronger than those seen following Smo loss of function in retinal progenitors. The quality of the data is excellent, analysis thorough, and results convincing. This is an important new contribution to our understanding of the regulation of retinal patterning and neurogenesis.

Comments for the author

However, additional data would tie up some of the loose ends in the paper. Specific suggestions for improvement are listed below:

1. The disruption of naso-temporal patterning seen at E13 in Figure 4 is striking, particularly the fact that segregation of Foxd1 and Foxg1 expression is not affected at E10, despite efficient loss of Smo function at this stage. The authors speculate that Smo activity is not required for establishing naso-temporal patterning but is required for its maintenance at later stages. This could be easily tested by analyzing Foxd1 and Foxg1 in the Chx10-Cre mutant lines.
2. The expanded temporal window for RGC and cone generation suggests that temporal patterning in retinal progenitors has been altered. However, no information is provided about the mechanism here. One way to provide insight here would be to analyze RPCs for expression of genes linked to early (e.g. Nr2f1, Meis1, Atoh7) and late-stage (e.g. Nfia/b/x, Sox8) temporal identity at ages after E17, when the temporal transition between these states occurs. It would also be helpful to know when generation of these early-born cells ceases. Do the data here just reflect a minor shift in temporal patterning, or does cone and RGC generation continue until postnatal stages?

Minor point: Gene names should be italicized when mRNA encoded by the gene is discussed (e.g. in situ hybridization experiments).

Reviewer 2

Advance summary and potential significance to field

In this study, the authors present a comprehensive analysis of Shh signaling during different stages of retinal development, using temporally and spatially controlled deletion of Smoothened (Smo), an essential Hedgehog pathway transducer. They propose that this approach uncovers previously unrecognized requirements for Shh signaling in establishing optic nerve head identity and maintaining nasal-temporal retinal polarity. The authors also report that Shh signaling coordinates neurogenesis by sustaining the retinal progenitor pool while regulating early versus late progenitor competence, thereby ensuring the generation of appropriate proportions of retinal cell types. Overall, the authors conclude that their work provides new insights into how morphogen signaling is repurposed across developmental stages to orchestrate complex tissue architecture.

Comments for the author

© 2026. Published by The Company of Biologists under the terms of the Creative Commons Attribution License (https://creativecommons.org/licenses/by/4.0/).

This is a carefully executed study that analyzes the observed phenotypes primarily in Rax-SmoCKO embryos. However, in contrast to the author claims, the novelty of the findings appears limited. Most of the functions they described for Shh signaling in eye development have already been reported, and the main contribution here is the compilation of these roles into a single manuscript.

Major points

1. The section comparing the phenotypes obtained with Chx10-Cre and Rax-Cre drivers spans nearly a page of results. Since the temporal activity of both drivers has been extensively studied and is well known, this description could be considerably shortened. The manuscript would benefit from devoting more space to novel aspects of the study.
2. The most potentially original contribution is the proposed role of Shh in regulating temporal retinal competence. Surprisingly, the authors do not take advantage of the Chx10-Cre driver to address this question directly. Demonstrating whether the phenotype is reproduced in Chx10-SmoCKO would clarify whether the effect reflects a direct role of Shh in competence or is secondary to earlier retinal defects. This experiment should be performed. The following reference should also be considered when discussing neurogenesis: PMID: 15253932.
3. Another potentially interesting aspect, the influence of Shh on astrocytes and blood vessel formation, is treated only superficially. The claim that astrocyte "numbers" increase (p.16) requires proper quantification, e.g. counting Sox9-positive cells. Alternative explanations should also be considered, such as defective astrocyte dispersion or reactive changes leading to increased GFAP expression and ramification. The study in PMID: 26116667 is relevant here and should be discussed.
4. The statement that the study uncovers "previously unrecognized requirements for Shh signaling in specifying optic nerve head identity and maintaining nasal-temporal polarity" (abstract, results, discussion) is inaccurate. Prior work by Dakubo et al. and Morcillo et al. (already cited in the manuscript) provides clear evidence that Shh is required for specification of the optic nerve head. In fact, the reference to Morcillo et al. is misreferenced: this study does not show a role in optic fissure closure but rather in optic nerve head specification.

Minor issues

5. The claim that "cone photoreceptors displayed altered morphologies with shortened axons and outer segments (Fig. 7E)" is not supported by the images shown. A higher-magnification image is required, particularly to assess axon length.
6. Similarly, the statement that "some of these were ectopically positioned in the ONL and displayed signs of abnormal polarity" should be supported by immunostaining for polarity markers.

In conclusion, I recommend that the authors substantially revise the manuscript. They should reduce emphasis on findings that are confirmatory, with appropriate citation of prior literature, and more accurately describe the novelty of their work. Greater attention should be devoted to the potentially original aspects, specifically the regulation of temporal competence and astrocyte/blood vessel development, ideally through additional experiments. This would allow a more definitive assessment of whether the observed defects reflect a direct requirement for Shh signaling or are secondary consequences of early retinal abnormalities

<u>Reviewer 3</u>

Advance summary and potential significance to field

This well-executed study explores the roles of Hedgehog signaling in ocular development by comparing two conditional Cre drivers that differ in the timing of Smo KO (an essential Hedgehog pathway activator). This approach clarifies when and where Hedgehog signaling is essential during early eye patterning and retinal development and uncovers previously unrecognised functions of this pathway in the optic nerve head specification, retinal patterning, and in the temporal competence of retinal progenitor cells. This study builds on the observations of several labs, especially regarding the role of Hedgehog in the retina, and the authors are very careful to highlight the novel functions of Hedgehog in this context, which adds value to the field. However, as a descriptive study, it could make a greater impact if it included more investigation, particularly into the temporal competence function.

© 2026. Published by The Company of Biologists under the terms of the Creative Commons Attribution License (https://creativecommons.org/licenses/by/4.0/).

The major strengths lie in the elegant two-Cre approach and the use of drivers that produce full Shh and Smo KO in the retina, which reduces potential confounds in interpreting previous studies that used region- or mosaic-Cre to induce Shh and Smo KO. The use of the early Cre driver and the clarity in how they define KO and WT tissues also reveals non-cell autonomous functions of Hedgehog signaling in the development of the anterior segment, prompting questions for future research. The paper is exceptionally clear and accessible, even for non-specialists. The authors present very convincing evidence that in SmoKO retinas, there is a temporal shift in early retinal cell type production—the period of RGC and cone production is extended. This clearly demonstrates the role of Shh signaling in temporal competence. Overall, the experiments are rigorous and well-supported by compelling histology images.

Comments for the author

The study does not probe how Shh regulates temporal competence. For example, are competence factors (beyond Atoh7) altered at the protein level? This would enhance the study's impact.
It also remains unresolved whether later changes in cell fate reflect a direct requirement for Shh in the retina or could, in part, be a consequence of earlier disruptions in eye patterning. Could the authors expand on this in the discussion?
Clarification of the source of Hedgehog ligand, particularly in the early ocular patterning stages. Is the relevant ligand long-range from the midline, or relayed through intermediate tissues?
Could any of the phenotypes reflect Hh-independent Smo activity? While unlikely, the authors might briefly address this possibility.

First revision

Author response to reviewers' comments

Comments from the Reviewers:

Reviewer 1: SUMMARY OF THE ADVANCE MADE IN THIS PAPER AND ITS POTENTIAL SIGNIFICANCE TO THE FIELD

This manuscript describes the effects of conditional loss of function of Smoothened (Smo), and by consequence Shh/Ihh signaling, in Rax-positive ocular neuroepithelial cells. While previous studies had characterized the effects of disrupting Smo in Vsx2-positive retinal progenitor cells, the current study describes the phenotype of a substantially earlier loss of function. This leads to dramatic changes in retinal patterning, including conversion of optic nerve into neuroretina, breakdown of anterior-posterior patterning, and loss of ciliary margin. Precocious neurogenesis and changes in temporal patterning are also observed, which are substantially stronger than those seen following Smo loss of function in retinal progenitors. The quality of the data is excellent, analysis thorough, and results convincing. This is an important new contribution to our understanding of the regulation of retinal patterning and neurogenesis.

We thank this reviewer for their support and thoughtful comments!

SUGGESTIONS TO AUTHORS

However, additional data would tie up some of the loose ends in the paper. Specific suggestions for improvement are listed below:

1. The disruption of naso-temporal patterning seen at E13 in Figure 4 is striking, particularly the fact that segregation of Foxd1 and Foxg1 expression is not affected at E10, despite efficient loss of Smo function at this stage. The authors speculate that Smo activity is not required for establishing naso-temporal patterning but is required for its maintenance at later stages. This could be easily tested by analyzing Foxd1 and Foxg1 in the Chx10-Cre mutant lines.

© 2026. Published by The Company of Biologists under the terms of the Creative Commons Attribution License (https://creativecommons.org/licenses/by/4.0/).

1.1 We directly tested this possibility as suggested. To this end, we performed RNAscope *in situ* hybridization on Chx10- Cre; SmoCKO mice and littermate controls using *FoxD1* (teal) and *FoxG1* (magenta) probes. As shown in the figure (new Suppl. Fig. 3), we do not observe defects in naso-temporal (N-T) patterning when using the Chx10-Cre driver. This result was unexpected, but several explanations could account for it:
-First, consistent with the normal patterning observed at E11, Shh signaling may not be required for the initial specification of the naso-temporal axis but instead during a specific early developmental window (E11–E12) for maintenance or refinement. In this model, Chx10-Cre; SmoCKO mice fail to exhibit a phenotype because *Smo* deletion occurs after this critical window, allowing N-T patterning to proceed normally.
-Second, it is possible that Chx10-Cre; SmoCKO mice have not yet manifested the phenotype by E13, or that incomplete recombination, given the known mosaicism of this line, preserves sufficient Shh signaling to maintain normal patterning.
-Finally, spatial differences in Cre expression may contribute. Rax-Cre is expressed in the optic nerve head (ONH), whereas Chx10-Cre is not. Deletion of *Smo* with Rax-Cre could alter ONH-derived signals or structural cues that indirectly influence the maintenance or refinement of N-T patterning, while Chx10-Cre leaves the ONH intact, permitting these processes to proceed normally. However, there is currently little direct evidence that the ONH itself functions as a signaling center instructing axis identity. Thus, this is speculation at this point.
In light of these new data, and to ensure a cautious and accurate interpretation, we have revised the text accordingly:

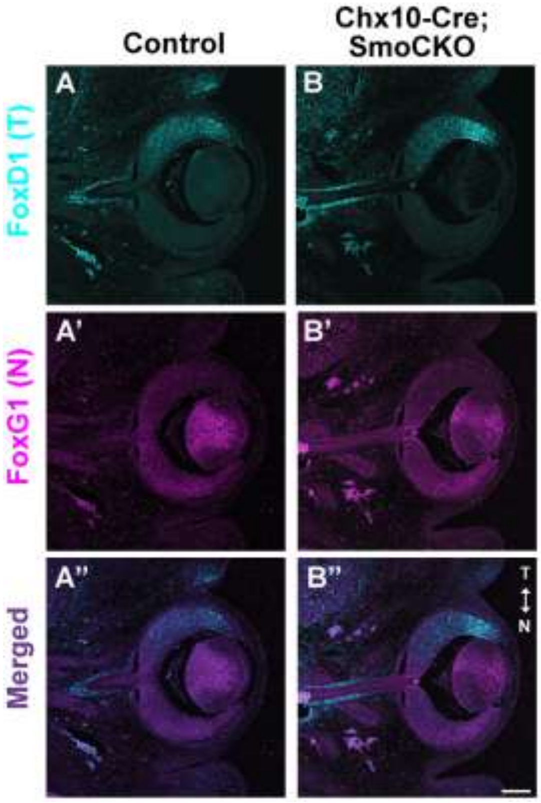

Results section:

"These findings suggest that while Shh signaling may be dispensable for early naso-temporal specification, it is required during a defined developmental window for maintenance and refinement of this axis. To further validate this hypothesis, we also investigated the expression patterns of Foxg1 and Foxd1 in Chx10-SmoCKO retinas. In contrast to Rax-Cre; SmoCKO, Chx10-Cre; SmoCKO retinas did not exhibit detectable alterations in nasal-temporal patterning at E13 (Suppl. Fig. 3)."

Discussion section:

© 2026. Published by The Company of Biologists under the terms of the Creative Commons Attribution License (https://creativecommons.org/licenses/by/4.0/).

"Importantly, we did not detect nasal-temporal defects in Chx10-Cre; SmoCKO retinas at E13. This absence of phenotype may reflect temporal differences in Cre activity and/or the mosaicism of the Chx10-Cre line, which could preserve sufficient Shh signaling to maintain axis organization."

1.2. The expanded temporal window for RGC and cone generation suggests that temporal patterning in retinal progenitors has been altered. However, no information is provided about the mechanism here. One way to provide insight here would be to analyze RPCs for expression of genes linked to early (e.g. Nr2f1, Meis1, Atoh7) and late- stage (e.g. Nfia/b/x, Sox8) temporal identity at ages after E17, when the temporal transition between these states occurs. It would also be helpful to know when generation of these early-born cells ceases. Do the data here just reflect a minor shift in temporal patterning, or does cone and RGC generation continue until postnatal stages?

Minor point: Gene names should be italicized when mRNA encoded by the gene is discussed (e.g. in situ hybridization experiments).

As suggested, we examined the expression of an early RPC gene (FoxP1, PMID: 36924502) and a late progenitor gene (Ascl1, PMID: 21771810) at P1, a developmental stage at which RPCs are expected to have transitioned from early to late temporal identities. As described previously, FoxP1 levels are very low at this stage and not reliably quantifiable. However, in Rax-Cre; SmoCKO retinas, we detect a subset of cells with elevated FoxP1 that co-localizes with PCNA (arrows). Please, note that FoxP1 is also expressed in a subpopulation of RGCs (arrowheads). In contrast, although many late RPCs express Ascl1 in controls, Ascl1 levels are significantly reduced in PCNA+ cells of Rax-Cre; SmoCKO retinas (*P*-value= 0.007).

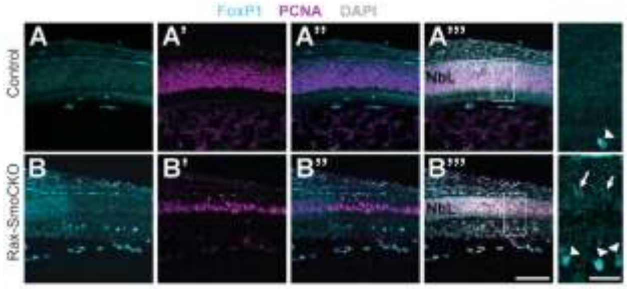

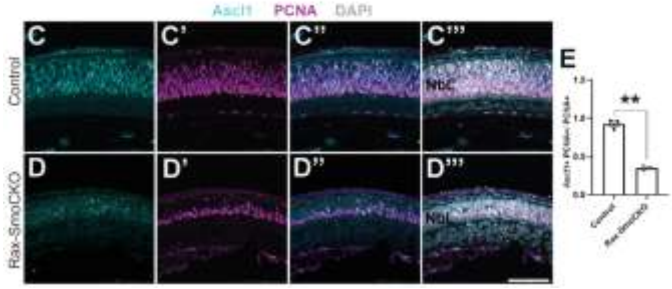

These findings are further supported by preliminary EdU birthdating experiments at P1, which suggest that cone photoreceptors are born in higher numbers at postnatal stages upon *Smo* ablation. We injected EdU at P1 and collected the retinas at P4. As expected, the total number of EdU+ cells was markedly reduced in Rax-Cre; SmoCKO retinas compared with controls, consistent with decreased progenitor proliferation. Despite this overall reduction in RPCs, we observed more EdU+ S-Opsin+ cells in Rax-Cre; SmoCKO retinas than in controls (arrows in bottom panels). Very few EdU+ RGCs (RBPMS+) were detected in Rax-Cre; SmoCKO retinas, whereas none were observed in controls.

© 2026. Published by The Company of Biologists under the terms of the Creative Commons Attribution License (https://creativecommons.org/licenses/by/4.0/).

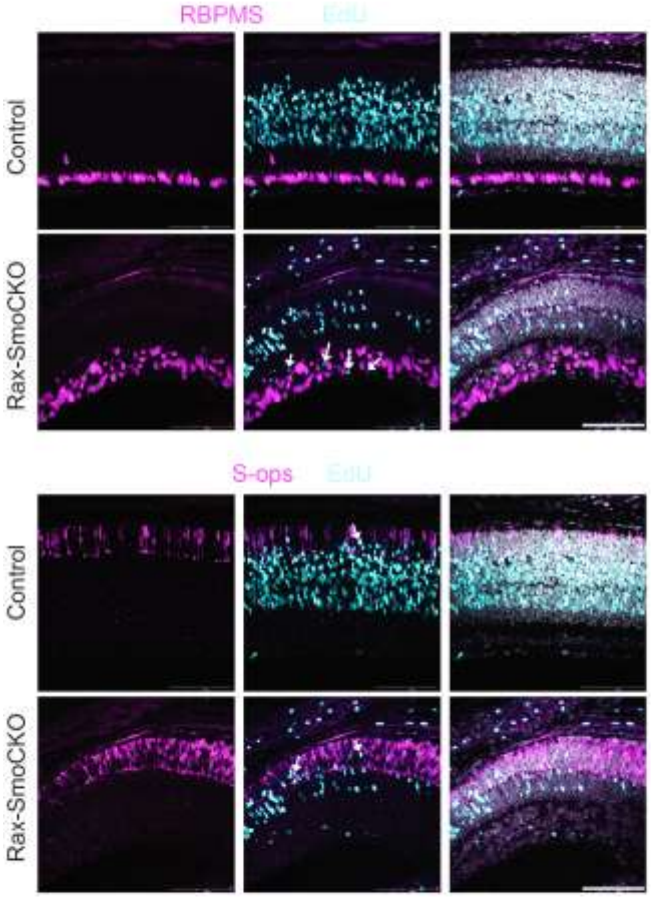

Interestingly, EdU+ cells were present in the GCL (arrows, EdU+ RBPMS- cells), but are absent from this layer in controls, potentially representing displaced amacrine populations. The GCL contains a disproportionate number of amacrine subtypes born at earlier developmental windows (e.g., starburst and dopaminergic amacrines), although displaced amacrines as a whole are not exclusively early- born. Thus, the presence of EdU+ RBPMS-cells in the GCL is also consistent with an extended temporal competence state. These observations, together with the increase in FoxP1 and the reduction in Ascl1 at P1, suggest that the temporal effects of *Smo* deletion may extend beyond E17. Future studies will build on these findings to investigate the molecular mechanisms driving altered progenitor competence.
Due to emerging breeding limitations associated with the SmoCKO line (small litter sizes and perinatal lethality), these experiments have limited sample numbers (4 eyes from 2 animals), and are not included in the manuscript. To enhance breeding, the line was previously backcrossed for five generations with CD-1 mice (see Materials and Methods). However, over time, these issues have reemerged, and additional backcrossing may be required for future experiments, but requires 2 years, and it's not feasible for these revisions.

The relevant sections of the manuscript now read:

"In order to assess whether RPC competence was affected in Rax-SmoCKO retinas, we labeled progenitors with 5-ethynyl-2'-deoxyuridine (EdU) at E17, when the progenitors in the central retina have normally transitioned to a late competence stage and lost their ability to generate early cell types such as RGCs and cone photoreceptors (Young, 1985, Reese and Colello, 1992, Brzezinski et al., 2012, Clark et al., 2019). We assessed the fate identities the central retina EdU+ cells at P1. As expected, EdU+ RBPMS+ RGCs were absent in control retinas (Fig 9). Strikingly, however, numerous EdU+ RBPMS+ cells were observed in Rax-SmoCKO eyes (p=0.007), along with a significant increase in EdU+ S-opsin+ cones (p=0.0008, Fig. 9). These findings indicate that loss of Smo extends the window of early progenitor competence, allowing for the continued production of early-born retinal neurons beyond their normal developmental timeframe.

To further confirm this hypothesis, we also stained P1 retinas with the early RPC marker, FoxP1

© 2026. Published by The Company of Biologists under the terms of the Creative Commons Attribution License (https://creativecommons.org/licenses/by/4.0/).

(Zhang et al., 2023a) as well as the late PRC marker, Ascl1 (Brzezinski et al., 2011). As reported previously, FoxP1 expression is very low at this stage and not reliably quantifiable. However, in Rax-Cre; SmoCKO retinas, we detected a subset of cells with increased FoxP1 immunoreactivity relative to controls, and these cells co-localize with PCNA (Suppl. Fig. 10, arrows), indicating expression within proliferating progenitors. Notably, FoxP1 is also expressed in a subpopulation of RGCs (arrowheads), consistent with prior reports. These are easily distinguished by their position in the GCL. In contrast, whereas many late RPCs express Ascl1 in control retinas, Ascl1 immunoreactivity is significantly reduced in PCNA+ cells of Rax-Cre; SmoCKO retinas (Suppl. Fig. 10). Together, these data support a shift in progenitor state, characterized by relatively elevated early RPC marker expression and diminished late RPC features following Smo deletion."

We have revised the text to make sure that all references to mRNAs are italicized. Thank you for catching this!

Reviewer 2: SUMMARY OF THE ADVANCE MADE IN THIS PAPER AND ITS POTENTIAL SIGNIFICANCE TO THE FIELD

In this study, the authors present a comprehensive analysis of Shh signaling during different stages of retinal development, using temporally and spatially controlled deletion of Smoothened (Smo), an essential Hedgehog pathway transducer. They propose that this approach uncovers previously unrecognized requirements for Shh signaling in establishing optic nerve head identity and maintaining nasal-temporal retinal polarity. The authors also report that Shh signaling coordinates neurogenesis by sustaining the retinal progenitor pool while regulating early versus late progenitor competence, thereby ensuring the generation of appropriate proportions of retinal cell types.
Overall, the authors conclude that their work provides new insights into how morphogen signaling is repurposed across developmental stages to orchestrate complex tissue architecture.

We thank the reviewer for the thoughtful and insightful comments. Addressing these suggestions has strengthened and improved the manuscript. We have carefully considered and addressed all points raised.

SUGGESTIONS TO AUTHORS

This is a carefully executed study that analyzes the observed phenotypes primarily in Rax-SmoCKO embryos. However, in contrast to the author claims, the novelty of the findings appears limited. Most of the functions they described for Shh signaling in eye development have already been reported, and the main contribution here is the compilation of these roles into a single manuscript.

We thank the reviewer for this thoughtful comment. We fully acknowledge that the roles of Shh signaling in eye development have been extensively investigated, including foundational studies from the groups of Valerie Wallace, Paola Bovolenta, and many others, and we recognize this important body of work in the manuscript. While our study builds directly on these prior findings, we believe it also adds new insights. In particular, the direct comparison of distinct Cre drivers provides important temporal and spatial resolution of Shh pathway function and clarifies how phenotypic outcomes depend on the timing and location of *Smo* deletion. Notably, despite the extensive literature on Shh signaling in ocular development, conditional inactivation of the pathway using an early eye field driver such as Rax-Cre (active from approximately E8) has not previously been reported in mice that survive to adulthood, allowing analysis of both developmental and postnatal consequences of early pathway disruption. We also report novel effects on nasal- temporal patterning, temporal competence regulation, as well as anterior segment and astrocyte development that have not been systematically examined in this context. Together, these results complement and extend previous studies of Shh signaling in the developing eye..

Major points
2.1. The section comparing the phenotypes obtained with Chx10-Cre and Rax-Cre drivers spans nearly a page of results. Since the temporal activity of both drivers has been extensively

© 2026. Published by The Company of Biologists under the terms of the Creative Commons Attribution License (https://creativecommons.org/licenses/by/4.0/).

studied and is well known, this description could be considerably shortened. The manuscript would benefit from devoting more space to novel aspects of the study.

Thank you for the suggestion, we agree. We have substantially shorted this section and now reads:

"Smoothened (Smo) is as a critical transducer in the Shh signaling pathway, conveying signals from the Shh ligand to downstream effectors that regulate gene expression (Alcedo et al., 1996, van den Heuvel and Ingham, 1996) (Fig. 1A). To investigate the effects of Shh pathway disruption in the developing retina, we employed a conditional loss-of-function approach by using a Smo$^{CKO/CKO}$ allele (Long et al., 2001) crossed with either Chx10-Cre or Rax-Cre drivers. Chx10-Cre is expressed in retinal progenitor cells (RPCs) starting at embryonic day (E) 10 in a mosaic pattern (Rowan and Cepko, 2004) (Fig. 1B). In contrast, Rax is expressed earlier, starting at E7.5 (Furukawa et al., 1997, La Torre et al., 2015) and the Rax-Cre is active from E8 in the ventral thalamus/hypothalamus and optic vesicle, cup and stalk, and all subsequently arising tissues such as the RPE, ONH, ciliary body, and all retinal cell types (Bosze et al., 2020) (Fig. 1B). RNAscope in situ hybridization (ISH) confirmed Smo ablation. At E10, only Rax-SmoCKO showed complete ablation; Chx10-SmoCKO retina remained unaffected. By E13.5, both mutants lost Smo throughout the retina, but Chx10-SmoCKO RPCs retained mosaic expression, while Rax-SmoCKO also lost Smo in the RPE, ONH, and optic stalk, with reduced Gli1 and Ptch1 (Suppl. Fig. 1). Thus, earlier and broader Rax-Cre activity predicts distinct defects in optic cup morphogenesis and patterning. In Chx10-SmoCKO, residual wild-type clones may partially mitigate some effects, potentially masking certain early Shh requirements."

2.2 The most potentially original contribution is the proposed role of Shh in regulating temporal retinal competence. Surprisingly, the authors do not take advantage of the Chx10-Cre driver to address this question directly. Demonstrating whether the phenotype is reproduced in Chx10-SmoCKO would clarify whether the effect reflects a direct role of Shh in competence or is secondary to earlier retinal defects. This experiment should be performed. The following reference should also be considered when discussing neurogenesis: PMID: 15253932.

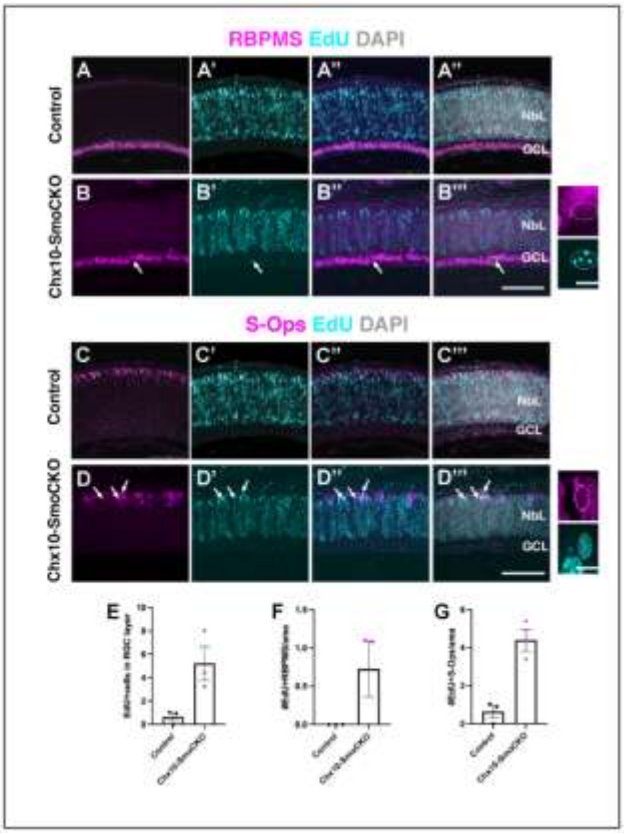

As suggested, we performed additional EdU birthdating experiments (Suppl. Fig. 11) to determine

© 2026. Published by The Company of Biologists under the terms of the Creative Commons Attribution License (https://creativecommons.org/licenses/by/4.0/).

whether the timing of neurogenesis is altered in Chx10-Cre; SmoCKO retinas. EdU was administered at P0, and retinas were collected at P2. By this stage, RPCs have largely transitioned to late competence states and are therefore expected to generate late-born cell types, but not RGCs or cone photoreceptors, whose production is largely complete by E16.
Consistent with this, we did not detect any EdU+ RBPMS+ RGCs in control retinas. In contrast, a small fraction of EdU+ RBPMS+ cells was observed in Chx10-Cre; SmoCKO samples (arrow in B-B’’’). Similarly, *Smo*-ablated retinas displayed an increased fraction of EdU+ S-Opsin+cones (C-D’’’ and G).
We also noted an overall increase in EdU+ cells within the GCL (Fig. 11E). Many of these cells were RBPMS-, suggesting they are displaced amacrine cells. Although displaced amacrines are not exclusively early-born, the GCL contains a disproportionate representation of amacrine subtypes generated during earlier developmental windows (*e.g.*, starburst and dopaminergic amacrines).
Thus, the presence of EdU+ RBPMS- cells in the GCL further supports the persistence of an extended temporal competence state in Chx10-Cre; SmoCKO retinas.
We have added this figure to the paper and now the relevant sections read:

“To determine whether the observed alterations in temporal neurogenesis were secondary to early developmental effects, given that Rax-Cre deletes Smo prior to the onset of neurogenesis, we examined temporal competence in Chx10- Cre; SmoCKO retinas, in which recombination occurs later in RPCs. EdU was administered at P0 and retinas were analyzed at P2.
As expected, no EdU+ RGCs were detected in control samples. In contrast, a small number of EdU+ RBPMS+ cells were observed in Smo-ablated retinas. Similarly, Chx10-Cre; SmoCKO retinas exhibited an increased proportion of EdU+ S-Opsin+ cone photoreceptors. We also observed an overall increase in EdU+ cells within the GCL. Many of these cells were RBPMS-, suggesting they correspond to displaced amacrine cells. Although displaced amacrines are not exclusively early-born, the GCL contains a disproportionate number of amacrine subtypes generated during earlier developmental windows, such as starburst and dopaminergic amacrines (Voinescu et al. 2010, Balasubramanian et al., 2015). Thus, this observation further supports the persistence of an extended temporal competence state in Chx10-Cre; SmoCKO retinas.”

2.3. Another potentially interesting aspect, the influence of Shh on astrocytes and blood vessel formation, is treated only superficially. The claim that astrocyte "numbers" increase (p.16) requires proper quantification, e.g. counting Sox9-positive cells. Alternative explanations should also be considered, such as defective astrocyte dispersion or reactive changes leading to increased GFAP expression and ramification. The study in PMID: 26116667 is relevant here and should be discussed.

We agree with this critique and have added a revised Supplementary Figure 7 that now includes Sox9 and GFAP immunolabeling experiments at P21 (A-B’’), along with quantifications. These experiments demonstrate a significant increase in astrocyte numbers (see Suppl. Fig. 7C).

© 2026. Published by The Company of Biologists under the terms of the Creative Commons Attribution License (https://creativecommons.org/licenses/by/4.0/).

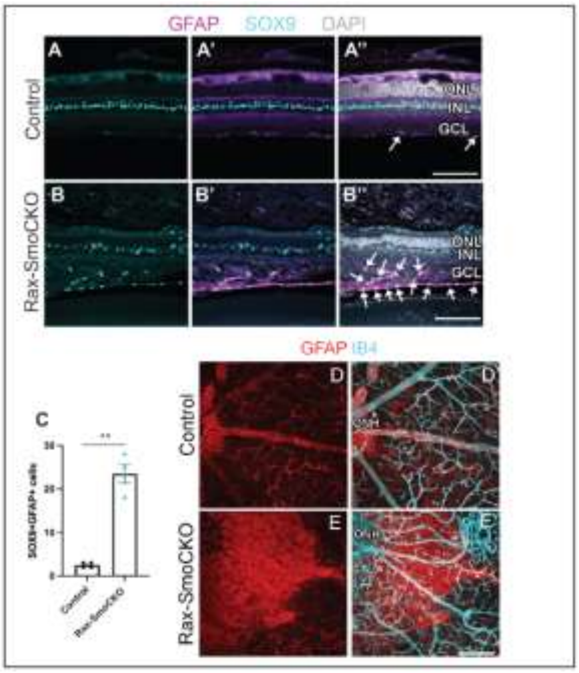

Preliminary data also indicate an increased number of EdU+ Sox9+ astrocytes at P0 (see figure below, arrows in bottom panels). As noted by the reviewer, this does not exclude other potential effects, including changes in ramification, morphology, or differentiation. The discussion has been updated to consider these possibilities, and we have included the important reference (Todd and Fisher, 2015) suggested by the reviewer.

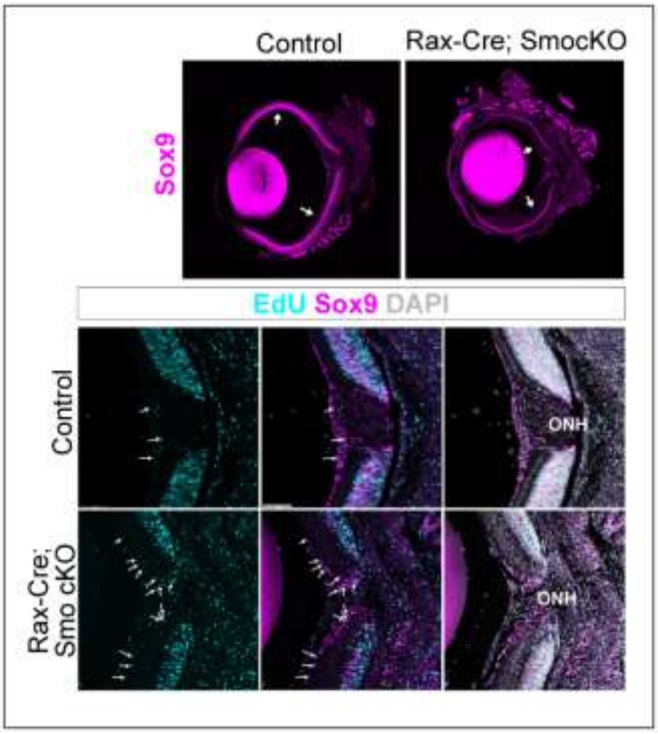

Now the relevant parts of the paper read:

"Finally, we also observed changes in retinal glial populations, including astrocytes. These findings further confirm a body of evidence that RGCs are required for the migration of retinal astrocytes, which in turn guide the migration and patterning of the adult retinal vasculature (Fruttiger et al., 1996, Burne and Raff, 1997, Wallace and Raff, 1999, Edwards et al., 2012, Paisley and Kay, 2021,

© 2026. Published by The Company of Biologists under the terms of the Creative Commons Attribution License (https://creativecommons.org/licenses/by/4.0/).

O'Sullivan et al., 2017). Thus, the increased number and altered distribution of RGCs we observe following Shh pathway disruption could indirectly influence astrocyte patterning and vascular development. In addition to these indirect effects, Shh signaling may also influence glial populations more directly, regulating processes such as migration or maturation. For instance, a previous study (Todd and Fisher, 2015) demonstrated that Hedgehog signaling regulates the Müller glia in the injured avian retina.
Similarly, in the developing cortex, astrocytes respond to Shh signals from neurons, which influence their morphology, functional properties, and interactions with synapses (Hill et al. 2019, Xie et al., 2022)".

2.4. The statement that the study uncovers "previously unrecognized requirements for Shh signaling in specifying optic nerve head identity and maintaining nasal-temporal polarity" (abstract, results, discussion) is inaccurate. Prior work by Dakubo et al. and Morcillo et al. (already cited in the manuscript) provides clear evidence that Shh is required for specification of the optic nerve head. In fact, the reference to Morcillo et al. is misreferenced: this study does not show a role in optic fissure closure but rather in optic nerve head specification.

Thanks for this important comment. We are well aware of the studies by Dakubo et al. and Morcillo et al., and we agree that they provide clear evidence supporting a role for Shh signaling in ONH specification. Our intention was not to overlook or diminish these prior findings.
We have now thoroughly revised the manuscript to:

1. Remove the phrase "previously unrecognized requirements" where it overstated novelty. And revise the whole text similarly
2. Correct the citation of Morcillo et al., which indeed addresses ONH specification.
3. Clarify that our findings extend and refine previous work by defining the temporal and spatial requirements of Shh signaling in this context, rather than identifying the role *de novo*.

We appreciate this reviewer's careful reading and believe the revised text now more accurately reflects the foundational contributions of these studies while clearly positioning our work within that framework.

Minor issues

2.5. The claim that "cone photoreceptors displayed altered morphologies with shortened axons and outer segments (Fig. 7E)" is not supported by the images shown. A higher-magnification image is required, particularly to assess axon length.

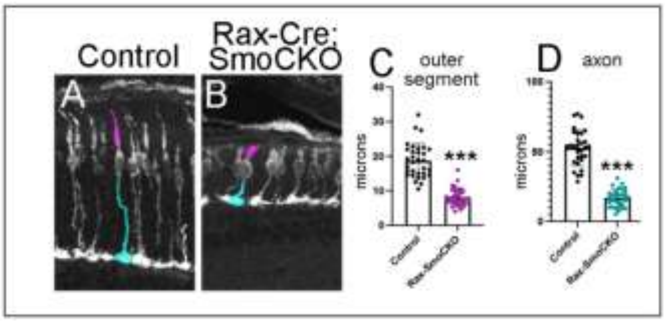

As suggested, we have taken higher confocal magnifications to show the shorter axonal length and outer segments of the Rax-Cre; SmoCKO axons. We have also quantified axonal and outer segment lengths, and they are both statistically shorter compared to controls. The shorter axonal length is not very surprising, as the whole ONL is much thinner, but the different outer segments may indicate broader morphological defects. This is now added to the manuscript as the new Supplementary Fig. 8.

2.6. Similarly, the statement that "some of these were ectopically positioned in the ONL and displayed signs of abnormal polarity" should be supported by immunostaining for polarity markers.
We thank the reviewer for the suggestion. We did not intend to assume that MG polarity is disrupted; rather, we wanted to point out that some MG nuclei are oriented "side-ways" relative to the typical tear-drop shape seen in controls. To clarify this point, we have updated the text

© 2026. Published by The Company of Biologists under the terms of the Creative Commons Attribution License (https://creativecommons.org/licenses/by/4.0/).

accordingly and added delineations of MG nuclei in Fig. 8 to better illustrate the observed nuclear orientation, as shown below. Now the relevant part of the results reads:

"We also observed a subset of MG cells ectopically displaced into the ONL. In addition, MG nuclei exhibited altered morphology, frequently oriented orthogonal to the retinal layers (Fig. 7J, arrows), instead of the tear-drop configuration characteristic of controls (Fig. 7H)."

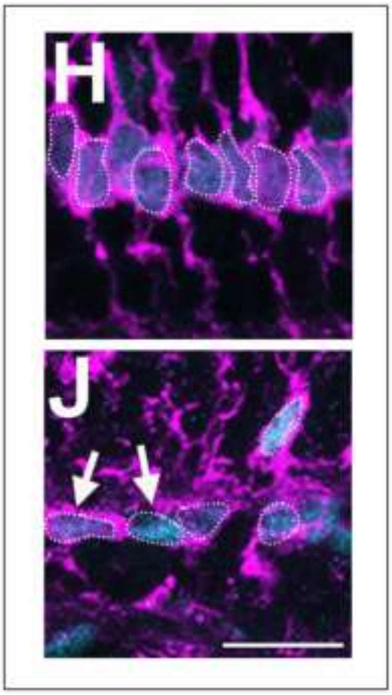

In conclusion, I recommend that the authors substantially revise the manuscript. They should reduce emphasis on findings that are confirmatory, with appropriate citation of prior literature, and more accurately describe the novelty of their work. Greater attention should be devoted to the potentially original aspects, specifically the regulation of temporal competence and astrocyte/blood vessel development, ideally through additional experiments. This would allow a more definitive assessment of whether the observed defects reflect a direct requirement for Shh signaling or are secondary consequences of early retinal abnormalities.

We thank the reviewer for this thoughtful feedback. The manuscript has been substantially revised to appropriately cite prior work and clarify the novelty of our study, with greater emphasis on the regulation of temporal competence and astrocyte/blood vessel development as potentially original aspects. These changes ensure that our findings are accurately contextualized within the existing literature.

Reviewer 3: SUMMARY OF THE ADVANCE MADE IN THIS PAPER AND ITS POTENTIAL SIGNIFICANCE TO THE FIELD

This well-executed study explores the roles of Hedgehog signaling in ocular development by comparing two conditional Cre drivers that differ in the timing of Smo KO (an essential Hedgehog pathway activator). This approach clarifies when and where Hedgehog signaling is essential during early eye patterning and retinal development and uncovers previously unrecognised functions of this pathway in the optic nerve head specification, retinal patterning, and in the temporal competence of retinal progenitor cells. This study builds on the observations of several labs, especially regarding the role of Hedgehog in the retina, and the authors are very careful to highlight the novel functions of Hedgehog in this context, which adds value to the field. However, as a descriptive study, it could make a greater impact if it included more investigation, particularly into the temporal competence function.

The major strengths lie in the elegant two-Cre approach and the use of drivers that produce full

© 2026. Published by The Company of Biologists under the terms of the Creative Commons Attribution License (https://creativecommons.org/licenses/by/4.0/).

Shh and Smo KO in the retina, which reduces potential confounds in interpreting previous studies that used region- or mosaic-Cre to induce Shh and Smo KO. The use of the early Cre driver and the clarity in how they define KO and WT tissues also reveals non-cell autonomous functions of Hedgehog signaling in the development of the anterior segment, prompting questions for future research. The paper is exceptionally clear and accessible, even for non-specialists. The authors present very convincing evidence that in SmoKO retinas, there is a temporal shift in early retinal cell type production—the period of RGC and cone production is extended. This clearly demonstrates the role of Shh signaling in temporal competence. Overall, the experiments are rigorous and well-supported by compelling histology images.

We thank this reviewer for their insightful comments!

SUGGESTIONS TO AUTHORS

3.1 The study does not probe how Shh regulates temporal competence. For example, are competence factors (beyond Atoh7) altered at the protein level? This would enhance the study's impact.

We thank the reviewer for this helpful suggestion. As this comment overlaps with a point raised by Reviewer 1, we refer the reviewer to our response to Reviewer 1, point 1.2, where this issue is discussed in detail.

3.2 It also remains unresolved whether later changes in cell fate reflect a direct requirement for Shh in the retina or could, in part, be a consequence of earlier disruptions in eye patterning. Could the authors expand on this in the discussion?

We thank the reviewer for this comment. This point is closely related to the concern raised by Reviewer 2 (point 2.2); please see our response there.

3.3 Clarification of the source of Hedgehog ligand, particularly in the early ocular patterning stages. Is the relevant ligand long-range from the midline, or relayed through intermediate tissues?

We agree that the source and mode of Hedgehog ligand delivery during early ocular patterning is an important question. During early forebrain and eye field development, Shh expressed in the ventral midline (prechordal plate and ventral forebrain) is well established as a critical regulator of optic vesicle separation and proximal-distal patterning. In this context, Shh is thought to act initially as a midline-derived signal that patterns adjacent neuroectoderm. As development proceeds, Shh is also expressed within RGCs, providing a secondary intra-retinal source of ligand.
Whether early ocular Shh activity reflects direct long-range diffusion from the midline or is propagated via intermediate tissues (*e.g.*, ventral forebrain or optic stalk) remains incompletely resolved. Our study does not directly distinguish between these possibilities. However, because the Chx10-Cre-mediated deletion of *Smo* occurs after optic cup formation and specifically in retinal progenitor cells, the phenotypes observed in this model are unlikely to result from altered midline-derived signaling during initial eye field patterning. Instead, they are consistent with a requirement for Shh pathway activity within the retina itself.

We have clarified this point in the Discussion.

3.4 Could any of the phenotypes reflect Hh-independent Smo activity? While unlikely, the authors might briefly address this possibility.

We also considered whether any phenotypes might reflect Hedgehog-independent functions of Smo. Although non- canonical Smo signaling has been reported in certain contexts, including GPCR-like signaling and regulation of cytoskeletal dynamics, most established roles for Smo in embryonic patterning are mediated through canonical Shh– Gli transcriptional signaling. Notably, the early ocular defects observed here, such as ventral patterning abnormalities and coloboma, are well-documented consequences of reduced Shh or Gli activity in vertebrate models. We therefore interpret the major phenotypes as reflecting impaired canonical pathway function, while acknowledging that we cannot formally exclude minor non-canonical contributions.

© 2026. Published by The Company of Biologists under the terms of the Creative Commons Attribution License (https://creativecommons.org/licenses/by/4.0/).

We have now acknowledged this possibility in the Discussion section.

Second decision letter

MS ID#: dev.205143R1

MS TITLE: Novel Roles of Sonic Hedgehog Signaling in Retinal Patterning and Neurogenesis During Mammalian Eye Development

AUTHORS: Miranda R. Krueger, Simranjeet K. Cheema, Sergi Simo, Edward M. Levine, Nadean L. Brown and Anna La Torre

Dear Dr La Torre,

I am happy to tell you that your manuscript has been accepted for publication in Development, pending our standard publication integrity checks.

Reviewer 1

Advance summary and potential significance to field

This manuscript describes the effects of conditional loss of function of Smoothened (Smo), and by consequence Shh/Ihh signaling, in Rax-positive ocular neuroepithelial cells. While previous studies had characterized the effects of disrupting Smo in Vsx2-positive retinal progenitor cells, the current study describes the phenotype of a substantially earlier loss of function. This leads to dramatic changes in retinal patterning, including conversion of optic nerve into neuroretina, breakdown of anterior-posterior patterning, and loss of ciliary margin. Precocious neurogenesis and changes in temporal patterning are also observed, which are substantially stronger than those seen following Smo loss of function in retinal progenitors. The extension of at least some aspects early-stage temporal identity into the early postnatal period seen following Smo loss of function is the most novel and exciting finding here.

Comments for the author

I appreciate the careful and extensive revisions the authors have provided. However, I would ask that the authors qualify their conclusions somewhat about the shift in temporal identity observed in the Smo knockout. Currently, this rests on the birthdating analysis of RGCs and cones, and expression of two early (Atoh7, Foxp1) and one late-ish (Ascl1) markers. A more complete analysis of both early and late-stage progenitor markers, along with birth dating of later-born cells, is necessary to definitively show this. This can be done with some fairly minor textual changes.

Reviewer 2

Advance summary and potential significance to field

The revised manuscript provides an interesting analysis of the temporal and spatial requirement of Shh signaling based on Rax-Cre and Chx10-Cre Smo-inactivation. The study highlights that Shh regulates retinal progenitor temporal competence, demonstrating that loss of Smo extends the window of early-born neuron production and partially uncoupling this effect from early morphogenetic defects. It also offers an integrated view of how Shh coordinates retinal neurogenesis, cell-type proportions, and glial development.

Comments for the author

© 2026. Published by The Company of Biologists under the terms of the Creative Commons Attribution License (https://creativecommons.org/licenses/by/4.0/).

The revised manuscript by Krueger et al. has been substantially improved and addresses many of the concerns raised in the initial review. The authors have incorporated new experimental data, particularly through the use of Chx10-Cre models and EdU birth-dating, which strengthen the conclusions regarding the temporal regulation of retinal neurogenesis by Shh signaling. Some mechanistic questions remain open (e.g. ligand source and signaling propagation, as raised by rev 3; astrocytes blood vessels interactions) but overall the main conclusions are well supported.

Reviewer 3

Advance summary and potential significance to field

I appreciate the authors' careful and thorough revision of the manuscript. In my previous review, I was largely supportive of the study. The main points I raised (as well as those of Reviewers 1 and 2), such as the need for the authors to provide more mechanistic insight into the temporal competence phenotype, have been satisfactorily addressed in this revision. Overall, the revisions have strengthened the manuscript and built upon previous work in the field. I have no further concerns and believe the study will engage readers interested in retinal patterning and histogenesis.

© 2026. Published by The Company of Biologists under the terms of the Creative Commons Attribution License (https://creativecommons.org/licenses/by/4.0/).
